# C118P Suppresses Gastric Cancer Growth via Promoting Autophagy–Lysosomal Degradation of RAB1A

**DOI:** 10.3390/pharmaceutics16121620

**Published:** 2024-12-21

**Authors:** Shihui Wei, Jing Zhang, Hai Wu, Zhengguang Liao, Zhengrui Liu, Yuhang Hou, Danyu Du, Jingwei Jiang, Li Sun, Shengtao Yuan, Mei Yang

**Affiliations:** New Drug Screening and Pharmacodynamics Evaluation Center, National Key Laboratory for Multi-Target Natural Drugs, China Pharmaceutical University, Nanjing 210009, China; wei_shihui@163.com (S.W.); zhangjing_991012@163.com (J.Z.); wuhai_cpu@163.com (H.W.); l18788843601@163.com (Z.L.); ccyfshengwu@163.com (Z.L.); hyh941591709@163.com (Y.H.); danyu_du@126.com (D.D.); jiangjingwei@126.com (J.J.); sunli@cpu.edu.cn (L.S.)

**Keywords:** C118P, RAB1A, mTOR, protein degradation, gastric cancer

## Abstract

**Background/Objectives**: Gastric cancer (GC) is the leading cause of cancer-related deaths worldwide. C118P, a microtubule inhibitor with anti-angiogenic and vascular-disrupting activities, was proven to be cytotoxic to various cancer cell lines. This study aimed to explore the anti-tumor effect of C118P against gastric cancer and identify its potential target. **Methods**: The MTT assay, colony formation assay, and EdU incorporation assay were used to evaluate the effect of C118P on GC cell proliferation. Cell cycle and cell apoptosis were measured using flow cytometry. Molecular docking, a microscale thermophoresis (MST) analysis, and the cellular thermal shift assay (CETSA) were used to investigate the binding of C118P to RAB1A. Autophagy-related effects were evaluated by using the MDC staining assay, immunofluorescence assay, and immunoblotting assay. The SGC-7901 cell line xenograft mouse model was used to confirm the anti-tumor efficacy of C118P. **Results**: C118P dramatically inhibited proliferation, induced G2/M cell cycle arrest, and triggered apoptosis in GC cell lines HGC-27 and SGC-7901. Mechanistically, C118P was demonstrated to bind with RAB1A and reduce the RAB1A protein level, accompanied by the inhibition of mTORC1 signaling. Moreover, C118P induced autophagosome formation and promoted RAB1A protein degradation in an autophagy–lysosomal-dependent manner. The in vivo study verified that C118P inhibits GC growth by inhibiting the RAB1A-mTOR axis. **Conclusions**: Our findings suggested that C118P inhibits GC growth by promoting the autophagy–lysosomal-dependent degradation of RAB1A and modulating mTOR C1 signaling. C118P shows potential as being a small molecule drug effective in the treatment of gastric cancer via targeting RAB1A.

## 1. Introduction

Gastric cancer (GC) is the most prevalent malignancy and the fifth leading cause of cancer deaths. Globally, 968,350 people are diagnosed with gastric cancer every year and 665,684 deaths were reported in 2022 worldwide [1]. Most cases occurred in Asia, particularly in China, accounting for more than 70% of the global case burden [2]. According to a statistical analysis of relevant data, the median survival of patients with advanced metastatic or unresectable gastric cancer is less than one year, and the five-year survival rate is less than 30% [3,4]. Recently, there have been significant advances in the treatment of gastric cancer. Besides surgery and systemic chemotherapy [5,6], immunotherapy (including nivolumab and pembrolizumab) [7,8] and targeted therapy including trastuzumab have proven to be effective against gastric cancer [9]. However, due to the molecular and phenotypic heterogeneity of gastric cancers [10,11], not all GC cases respond to these existing targeted therapies. Therefore, it is imperative to develop innovative targeted therapeutic approaches to treat gastric cancer.

Ras-related proteins (Rabs), the superfamily of tiny guanosine triphosphate (GTP)-binding proteins, regulate cell membrane transport and organelle function [12]. RAB1A is a member of the Rabs family that is highly conserved and mainly located in the endoplasmic reticulum, Golgi apparatus, endosomes, and cytosol [13,14]; it plays an essential role in nutrient sensing, signal transduction, cell migration, and autophagy regulation [15]. However, there is increasing evidence that the aberrant expression of RAB1A is involved in the development of human diseases, including cardiomyopathy [16], aspirin-exacerbated respiratory disease [17], sepsis [18], and, in particular, cancer [19]. Previous studies have shown that RAB1A is elevated in the progression of human cancers and associated with a poor prognosis in patients with cancers such as lung cancer [20], colorectal cancer [21], intrahepatic cholangiocarcinoma [22], gastric cancer [23], and bladder cancer [24]. In addition, accumulating evidence suggests that RAB1A acts as an oncogene by selectively activating the mTORC1 signaling pathway [25] and is mainly regulated by microRNAs [26,27], lncRNAs [28], and circRNAs [29]. While RAB1A is predicted to be a novel marker for the diagnosis, treatment, and prognosis of cancer patients in clinics, there is still a lack of molecularly targeted drugs for RAB1A.

C118P is a new class 1 drug for which a clinical phase I trial was approved in China. Recently, C118P was reported to possess vasculature disrupting and anti-angiogenic activities, and exhibits anti-tumor effects against hepatocellular carcinoma (HCC) [30]. Our previous data indicated that C118P might also have excellent prospects for the treatment of other solid tumors. However, the anti-tumor effect of C118P against gastric cancer and its underlying mechanism was unknown. In the present study, we evaluated the anti-gastric cancer effects of C118P in vitro and in vivo from several directions. We further elucidated the contribution of RAB1A to C118P-mediated anti-tumor effects against gastric cancer, which could provide guidance for the rational use of C118P in clinical trials.

## 2. Materials and Methods

### 2.1. Chemicals and Reagents

C118P (C18H17N2Na2O2P, purity of over 97.4%) was supplied by Nanjing Sanhome Pharmaceutical Co., Ltd. (Nanjing, China). C118P was dissolved in phosphate-buffered saline (100 mM) and stored at −80 °C. Taxol (TAX) was purchased from Yangtze River Pharmaceutical (Group) Co., Ltd. (#23081911, Taizhou, China). An RPMI-1640 medium and fetal bovine serum (FBS) were obtained from Gibco (Thermo Fisher Scientific, Waltham, MA, USA). 3-(4,5-Dimethylthiazol-2-yl)-2,5-diphenyltetrazolium bromide (MTT) and crystal violet were purchased from Sunshine Biotechnology Co., Ltd. (Nanjing, China). The EdU cell proliferation kit was provided by RiboBio Co., Ltd. (Guangzhou, China). The cell cycle detection kit was obtained from KeyGEN Biotech Co., Ltd. (Nanjing, China). The apoptosis detection kit (Annexin V-FITC/PI Staining) was purchased from Vazyme Biotech Co., Ltd. (Nanjing, China). The protease and phosphatase inhibitor complex was purchased from Beyotime Biotech (Shanghai, China). The autophagy staining assay kit with MDC was provided by KeyGEN Biotech Co., Ltd. (Nanjing, China). An antifade mounting medium with Hoechst 33342 was obtained from Beyotime Biotech (Shanghai, China). Cycloheximide (CHX), MG-132, 3-Methyladenine (3-MA), and chloroquine (CQ) were purchased from MedChemExpress (Monmouth Junction, NJ, USA).

### 2.2. Cell Culture

Human gastric cancer cell lines HGC-27 and SGC-7901 were purchased from the Cell Bank of Shanghai Institute for Biological Sciences, University of Chinese Academy of Sciences (Shanghai, China). HGC-27 and SGC-7901 were cultured in an RPMI-1640 medium containing 10% FBS and incubated in a humidified atmosphere with 5% CO_2_ at 37 °C. All cell lines were authenticated using a short tandem repeat analysis.

### 2.3. Cell Viability Assay

The effect of C118P treatment on gastric cancer cells was determined using the MTT assay. Cell suspensions were prepared and seeded into 96-well plates. After incubation for 24 h, cells were exposed to varying concentrations of C118P or Taxol for an additional 72 h. Subsequently, 20 μL of the MTT solution (0.5 mg/mL) was added to each well and incubated in the dark for another 4 h. The resulting formazan crystals were solubilized by adding 100 μL DMSO to each well after removing the supernatant. The absorbance was recorded at 570 nm using a Universal Microplate Reader (Infinite M100, Tecan, Crailsheim, Germany). The inhibition rate was determined using the following formula: Inhibition rate (%) = (1 − absorbance of the treated group/absorbance of the control group) × 100%.

### 2.4. Colony Formation Assay

The long-term effect of C118P treatment on cell proliferation was evaluated using the colony formation assay. Cell suspensions were prepared and seeded into 6-well plates. After incubation for 24 h, cells were treated with various concentrations of C118P or Taxol for another 24 h. Following this, the cells were cultured in a drug-free medium for an additional 7 days (HGC-27 cells) or 10 days (SGC-7901 cells) before fixation with 4% paraformaldehyde and staining with 0.5% crystal violet solution. Colony numbers were subsequently counted macroscopically.

### 2.5. EdU Cell Proliferation Assay

The short-term effect of C118P treatment on cell proliferation was assessed using the EdU incorporation assay. Cell suspensions were prepared and seeded into 96-well plates. After a 24 h incubation period, cells were exposed to varying concentrations of C118P or TAX for another 24 h. EdU staining was detected using the Cell-Light™ EdU Apollo488 In Vitro Kit according to the manufacturer’s instructions and observed using an inverted fluorescence microscope (Olympus, Tokyo, Japan).

### 2.6. Cell Cycle Analysis

The cell cycle distribution after C118P treatment was identified using propidium iodide (PI) staining. Following treatment with various concentrations of C118P or TAX for 24 h, cells were collected, and fixed overnight in 75% ethanol at 4 °C. After fixation, cells were rinsed with ice-cold PBS and incubated with the PI solution and RNase A for 30 min at 37 °C in the dark. The cell cycle profiles were analyzed using FACS-Calibur flow cytometry (BD Biosciences, San Jose, CA, USA).

### 2.7. Cell Apoptosis Analysis

The induction of apoptosis by C118P treatment was measured using Annexin V/PI staining. Following treatment with various concentrations of C118P or TAX for 24 h, cells were harvested using EDTA-free trypsin, and washed with ice-cold PBS. The Annexin V-FITC/PI apoptosis detection kit was applied following the manufacturer’s protocol. Apoptotic cell populations were quantified using FACS-Calibur flow cytometry (BD Biosciences, San Jose, CA, USA).

### 2.8. Molecular Docking

The files for RAB1A and its binding site were sourced from the scPDB database: http://bioinfo-pharma.u-strasbg.fr/scPDB/ (accessed on 4 July 2022). Molecular docking was performed using Autodock Vina (version 1.2.0), which employs a semi-flexible docking method and sorts the target proteins based on their docking binding energy.

### 2.9. Microscale Thermophoresis (MST) Analysis

The MST analysis was conducted using a NanoTemper Monolith NT.115 instrument (NanoTemper Technologies GmbH, Munich, Germany). The Monolith Protein Labeling Kit RED-NHS (L001) was sourced from NanoTemper Technologies (Watertown, MA, USA), while the Human Rab1a Recombinant protein (RAC6331) was obtained from Shanghai Chunmai Biotechnology Co., Ltd. (Shanghai, China). The Rab1a was labeled and purified using the monolith TMRED Red-NHS 2nd Generation protein labeling kit (NanoTemper Technologies GmbH). For NT.115 NanoTemper measurements, an infrared (IR) laser with a wavelength of 1480 nm was directed through a dichroic mirror onto the sample in a 25 µm diameter capillary. The IR laser is absorbed by the aqueous solution within the capillary and locally heats, and fluorescence is detected using a hot mirror at the same time. Thermophoresis of the protein in the presence of C118P at varying concentrations (ranging from 0.61 pM to 20 µM) was analyzed. All measurements were carried out at room temperature, with three replicate MST measurements performed per experimental set. A data analysis was conducted using the MO.Affinity Analysis v2.2.4 software (NanoTemper Technologies GmbH).

### 2.10. Immunoblotting Assay

Cells were washed with ice-cold PBS and lysed in a lysis buffer (Cell Signaling Technology, Beverly, MA, USA) containing protease and phosphatase inhibitors for total protein extraction. An immunoblotting analysis was performed as previously described [31]. The following primary antibodies were used and sourced from Cell Signaling Technology (Beverly, MA, USA): Anti-Cyclin B1 (#12231), anti-CDC2 (#77055), anti-p-CDC2 (#4539), anti-Cleaved Caspase-3 (#9664), anti-Cleaved Caspase-9 (#9509), anti-Cleaved PARP (#9541), anti-Bax (#2772), anti-Bcl-2 (#3498), anti-mTOR (#2972), anti-p-mTOR (#5536), anti-p-p70S6K (#9205), anti-4EBP1 (#9452), anti-p-4EBP1 (#2855), anti-LC3B (#3868), anti-Atg7 (#2631), anti-Atg5 (#2630), and anti-p62 (#8025). Anti-CDC25C (66912-1-Ig) and anti-RAB1A (11671-1-AP) were obtained from Proteintech Group (Wuhan, China). Anti-β-Actin (AC026), anti-LAMP1 (A23253), and anti-Beclin 1 (A7353) were purchased from ABclonal Biotechnology (Wuhan, China). Secondary antibodies, HRP-conjugated anti-mouse IgG (#7076) and anti-rabbit IgG (#7074), were sourced from Cell Signaling Technology (Beverly, MA, USA). Detection was carried out using enhanced chemiluminescence reagents (Millipore, Burlington, MA, USA), and imaging was performed with a Gel Doc system (Hercules, CA, USA).

### 2.11. Cellular Thermal Shift Assay

The cellular thermal shift assay (CETSA) was performed as previously described [32]. Briefly, SGC-7901 cells were seeded in 10 cm culture dishes and grown until 80–90% confluence. Then, the cells were harvested using 0.25% trypsin-EDTA (Servicebio, Wuhan, China) and washed with cold PBS. Afterward, the cells were resuspended in an RIPA lysis buffer containing a protease inhibitor cocktail and subjected to three freeze–thaw cycles using liquid nitrogen. The soluble fractions (lysates) were separated from the cell debris by centrifugation at 20,000× *g* for 20 min at 4 °C. Equal aliquots of the lysates were incubated with C118P (50 nM) or PBS for 3 h at 4 °C. The mixtures were then aliquoted into 100 μL samples and heated for 4 min at the designated temperatures (50–79.2 °C), followed by a 3 min cooling period at room temperature. After centrifugation at 20,000× *g* for 20 min at 4 °C, the soluble fractions were collected and analyzed using SDS-PAGE followed by an immunoblotting assay.

### 2.12. Quantitative Real-Time PCR (RT-qPCR)

Total cellular RNA was isolated using a TRIzol reagent (Vazyme Biotech, Nanjing, China) and reverse-transcribed using HiScript QRT SuperMix for qPCR (Vazyme Biotech, Nanjing, China). qPCR was performed with the AceQ qPCR SYBR Green Master Mix (High ROX Premixed) kit (Vazyme Biotech, Nanjing, China). Reactions were carried out on a StepOne™ Real-Time PCR System (Thermo Fisher Scientific, USA). The amount of mRNA for each gene was standardized using an internal control (18s rRNA), and the relative mRNA level of the treated group was based on the control group. The primers for amplification were as follows: RAB1A-Forward: 5′-GGGAACAAATGTGATCTGACCAC-3′, RAB1A-Reverse: 5′-GAAAGACTGTTCTACATTCGTTGC-3′, 18s rRNA-Forward: 5′-CTTTGGTCGCTCGCTCCTC-3′, and 18s rRNA-Reverse: 5′-CTG ACCGGGTTGGTTTTGAT-3′.

### 2.13. siRNA and Plasmids

RAB1A siRNA (siRAB1A#1 and siRAB1A#2) and non-targeting siRNA (siNC) were purchased from Shanghai GenePharma Co., Ltd. (Shanghai, China). The sequences of siRAB1A#1 and siRAB1A#2 are 5′-GGUGGAGGUUGCUGCUAAATT-3′ and 5′-CAAAGAAAGUAGUAGACUATT-3′. The sequence of siNC is 5′-UUCUCCGAACGUGUCACGUTT-3′. The plasmids of pLenti-CMV-RAB1A-Flag-GFP-Puro (PPL02635-4a) and pLenti-CMV-GFP-Puro (PE064) were obtained from Public Protein/Plasmid Library. Cell transfection was performed using lipofectamine 3000 (Thermo Fisher Scientific, USA), and the operation steps were based on the manufacturer’s protocol.

### 2.14. MDC Staining Assay

The effect of C118P treatment on cellular autophagy was detected via using monodansylcadaverine (MDC) staining. Cell suspensions were prepared and seeded into 12-well plates. After incubation for 24 h, the cells were treated with various concentrations of C118P or lysosomal inhibitor chloroquine (CQ) for another 24 h. The culture medium was then removed, and cells were processed using an MDC staining kit (KGATG001, KeyGEN BioTECH) according to the manufacturer’s instructions. Lysosomes in cells were visualized using an inverted fluorescence microscope (Olympus, Japan).

### 2.15. Immunofluorescence Assay

Gastric cancer cells were exposed to various concentrations of C118P or CQ for 24 h. After treatment, the cells were washed twice with ice-cold PBS, and then fixed in 4% paraformaldehyde for 30 min at room temperature. An immunofluorescence assay was conducted using the protocol as previously described in [33] with a primary antibody recognizing anti-LC3B (#3868, Cell Signaling Technology). These stained cells were observed and photographed with an Olympus FV1000 confocal laser scanning microscope (Olympus, Japan).

### 2.16. Xenograft Mouse Model of Gastric Cancer

Female BALB/c athymic nude mice (4–6 weeks) with body weight from 16 to 20 g were purchased from Zhejiang Vital River Laboratory Animal Technology Co., Ltd. (Jiaxing, China). SGC-7901 cells were suspended in a serum-free medium (5.0 × 10^6^ cells/200 μL) and subcutaneously injected into the right flank of mice. When the volume of tumors reached approximately 100 mm^3^, mice with analogous weight were randomly assigned to five groups (six mice per group) and treated with saline, C118P (50 mg/kg), C118P (75 mg/kg), C118P (100 mg/kg), and Taxol (15 mg/kg), respectively. C118P was administered intravenously at five doses per week for three consecutive weeks, while Taxol was given intravenously at two doses per week for 3 weeks. The control group received an equal volume of saline. The tumor volumes and body weight of mice were monitored throughout the experiment. After administration for 21 days, mice were sacrificed, then tumor tissues were harvested, photographed, and processed for further experiments. Tumor volume (TV) was calculated using the following formula: TV (mm^3^) = ab^2^/2, where a represents the longest diameter of the tumor, and b represents the shortest diameter.

### 2.17. Hematoxylin–Eosin (H&E) Staining

Fresh tumor tissues were fixed with 4% paraformaldehyde, dehydrated with graded ethanol, embedded in paraffin wax, and sectioned into 5 μm slices. These sections were stained with hematoxylin–eosin (H&E) to evaluate the changes in tumor histopathological characteristics under light microscopy.

### 2.18. Immunohistochemical Analysis

The tumor tissues of BALB/c nude mice were fixed in 4% paraformaldehyde for 24 h, followed by dehydration, paraffin embedding, and sectioning to a thickness of 4 μm. The immunohistochemistry analysis was performed with specific primary antibody anti-Ki67 (ab15580, Abcam, Cambridge, UK). The apoptotic cells induced by C118P or TAX in the tumor tissues were detected using the TUNEL Apoptosis Detection Kit (KeyGEN Biotech, Nanjing, China) according to the manufacturer’s instructions.

### 2.19. Statistical Analysis

Data are represented as the mean ± SD. Statistical significance was assessed by one-way ANOVA followed by Bonferroni post hoc comparisons using GraphPad Prism 8.0 software (La Jolla, CA, USA). The criterion for statistical significance was *p* < 0.05. The statistical significance is indicated as * *p* < 0.05, ** *p* < 0.01, *** *p* < 0.001, and # *p* < 0.0001.

## 3. Results

### 3.1. C118P Suppresses the Proliferation of GC Cells

**Figure 1:**
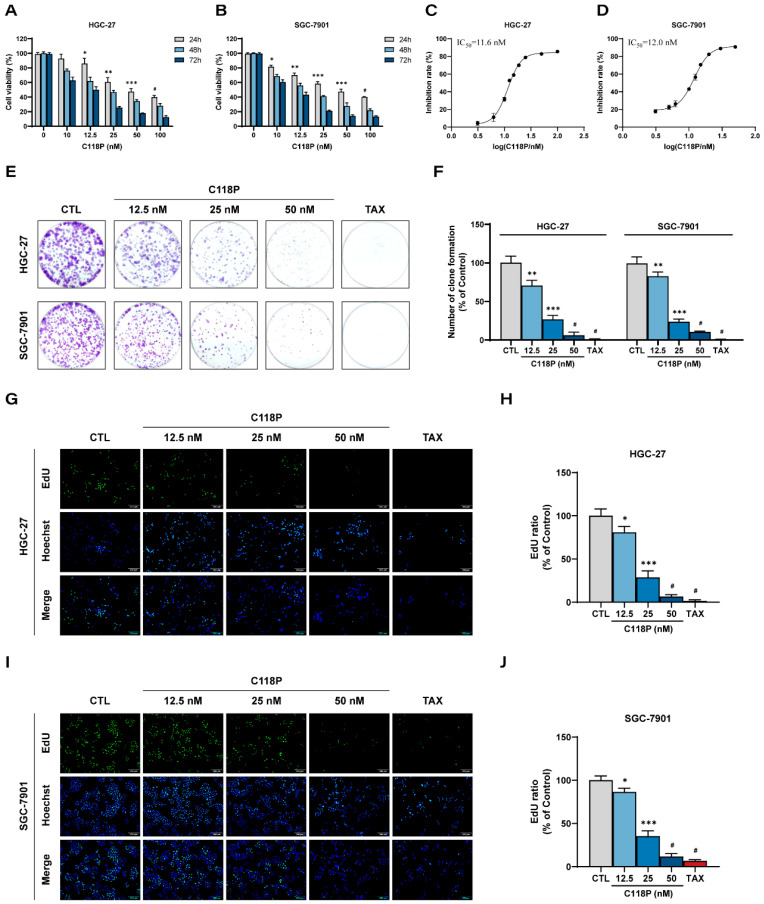
C118P inhibits cell proliferation in GC cell lines HGC-27 and SGC-7901. (**A**,**B**) Cell viability was determined with the MTT assay in HGC-27 (**A**) and SGC-7901 (**B**) cells treated with various concentrations (up to 100 nM) of C118P for 24 h, 48 h, and 72 h. (**C**,**D**) Cell proliferation curves of HGC-27 (**C**) and SGC-7901 (**D**) cells treated with various concentrations of C118P for 72 h were detected with the MTT assay. (**E**,**F**) Representative images (**E**) and summarized data (**F**) of the colony formation assay showing decreased cell density in GC cells treated with C118P (12.5, 25, 50 nM) for 7 days (HGC-27 cells) or 10 days (SGC-7901 cells). (**G**–**J**) The cell proliferation of HGC-27 (**G**,**H**) and SGC-7901 (**I**,**J**) cells treated with C118P (12.5, 25, 50 nM) for 24 h was tested with the EdU assay. Representative images (**G**,**I**) were photographed using an inverted fluorescence microscope and EdU ratios (**H**,**J**) were analyzed. Scale bar, 200 μm. Taxol (TAX, 50 nM) was used as the positive control. Data are represented as the mean ± SD of three independent experiments. * *p* < 0.05, ** *p* < 0.01, *** *p* < 0.001, ^#^ *p* < 0.0001 vs. control group.

C118P is a small molecule drug with the molecular formula C_18_H_17_N_2_Na_2_O_2_P and a molecular weight of 370.29. The chemical structure of C118P is shown in Supplementary Figure S1. To determine the fundamental anti-tumor role of C118P in gastric cancer, we first evaluated the anti-proliferative effect of C118P on GC cell lines using MTT assays, colony formation assays, and EdU staining. As shown in Figure 1A,B, C118P significantly decreased the cell viability of HGC-27 and SGC-7901 cells, as their IC_50_ values in response to C118P were (11.59 ± 0.48) nM and (12.0 ± 0.44) nM, respectively (Figure 1C,D). Additionally, Figure 1E,F show a dramatic decrease in cell colonies of both HGC-27 and SGC-7901 cells treated with C118P compared to that of controls, suggesting the potent anti-proliferative activity of C118P against GC cells (Figure 1E,F). Consistently, C118P significantly reduced the incorporation of the fluorescent nucleoside EdU into the GC cells’ DNA, indicative of DNA synthesis inhibition in C118P-treated GC cells (Figure 1G–J). Thus, findings from these tests all demonstrate that C118P exerts an anti-proliferative effect on GC cells that was comparable to the positive control Taxol.

### 3.2. C118P Induces G2/M Cell Cycle Arrest in GC Cells

**Figure 2:**
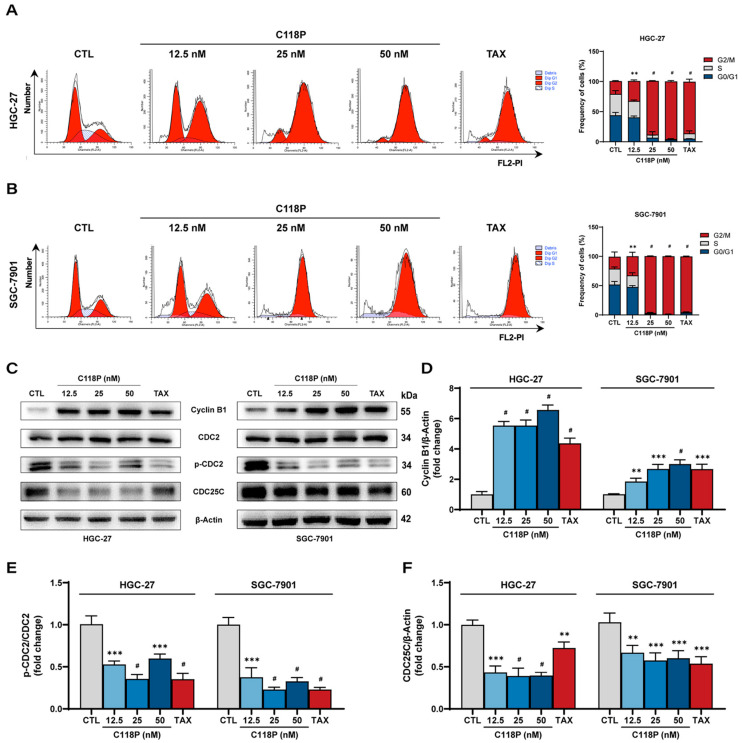
C118P induces cell cycle arrest in GC cell lines HGC-27 and SGC-7901. (**A**,**B**) Representative images (**left panel**) and summarized data (**right panel**) of the flow cytometry assay showing the cell cycle distribution of HGC-27 (**A**) and SGC-7901 (**B**) cells treated with C118P (12.5, 25, 50 nM) for 24 h. (**C**) Representative blot images showing protein expressions of Cyclin B1, p-CDC2, and CDC25C in HGC-27 and SGC-7901 cells treated with C118P (12.5, 25, 50 nM) for 24 h. (**D**–**F**) Summarized data showing protein expressions of Cyclin B1 (**D**), p-CDC2 (**E**), and CDC25C (**F**) in HGC-27 and SGC-7901 cells treated with C118P (12.5, 25, 50 nM) for 24 h. Taxol (TAX, 50 nM) was used as the positive control. Data are represented as the mean ± SD of three independent experiments. ** *p* < 0.01, *** *p* < 0.001, ^#^ *p* < 0.0001 vs. control group.

We next examined the effect of C118P on cell cycle distribution in GC cells using a flow cytometry assay (PI staining). Figure 2A,B show a significant increase in the proportion of HGC-27 and SGC-7901 cells in the G2/M phase following C118P treatment compared to that of controls, indicating that C118P induced cell cycle arrest in the G2/M phase. To clarify the mechanism of C118P on G2/M arrest, we assessed cell cycle checkpoints by detecting Cyclin B1, p-CDC2 (Tyr15), and CDC25C proteins using immunoblotting assays. Our data showed that the Cyclin B1 protein level increased while p-CDC2 (Tyr15) and CDC25C proteins’ level decreased in both HGC-27 and SGC-7901 cells after C118P treatment (Figure 2C–F). Thus, C118P induces G2/M-phase cell cycle arrest in GC cells through the CDC25C/Cyclin B1/CDC2 pathway.

### 3.3. C118P Induces Apoptosis in GC Cells

**Figure 3:**
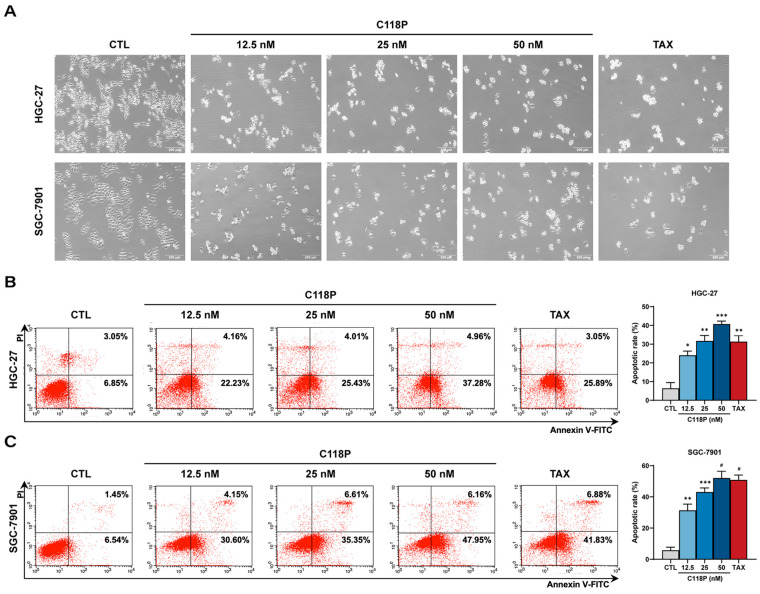

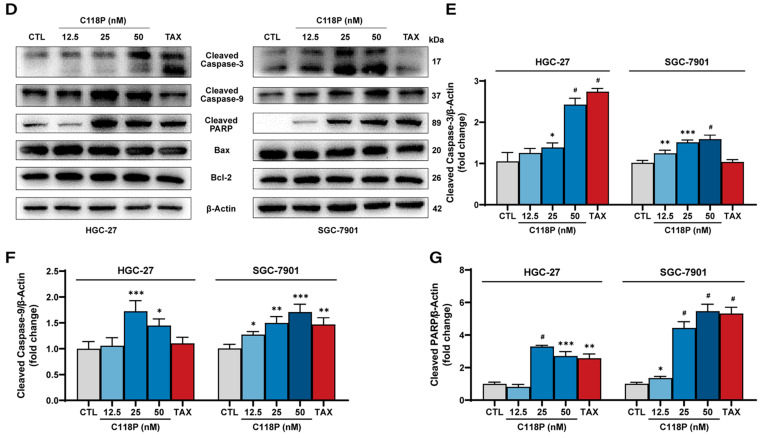
C118P induces apoptosis in GC cell lines HGC-27 and SGC-7901. (**A**) Representative images showing morphology changes in HGC-27 and SGC-7901 cells treated with C118P (12.5, 25, 50 nM) for 24 h. Scale bar, 200 μm. (**B**,**C**) Representative images (left panel) and summarized data (right panel) of the flow cytometry assay (Annexin V/PI) showing the apoptotic cells in HGC-27 (**B**) and SGC-7901 (**C**) cells treated with C118P (12.5, 25, 50 nM) for 24 h. (**D**) Representative immunoblotting images of Cleaved Caspase-3, Cleaved Caspase-9, Cleaved PARP, Bax, and Bcl-2 protein expression in HGC-27 and SGC-7901 cells treated with C118P (12.5, 25, 50 nM) for 24 h. (**E**–**G**) Summarized data of the immunoblotting assay showing increased protein expressions of Cleaved Caspase-3 (**E**), Cleaved Caspase-9 (**F**), and Cleaved PARP (**G**) in GC cell lines HGC-27 and SGC-7901 treated with C118P (12.5, 25, 50 nM) for 24 h. Taxol (TAX, 50 nM) was used as the positive control. Data are represented as the mean ± SD of three independent experiments. * *p* < 0.05, ** *p* < 0.01, *** *p* < 0.001, ^#^ *p* < 0.0001 vs. control group.

We further performed a series of assays to investigate whether C118P triggers apoptosis in GC cells, including morphological observation, Annexin V/PI staining, and an immunoblotting analysis. We initially observed that cells became rounded with contracted cytosolic morphology in both HGC-27 and SGC-7901 cell lines exposed to C118P (Figure 3A), indicating that C118P induces GC cell apoptosis. Indeed, Figure 3B,C show that C118P treatment significantly increased the apoptotic cell rate in a dose-dependent manner from (6.41 ± 2.14)% to (40.71 ± 1.15)% in HGC-27 cells, and (5.80 ± 1.35) % to (52.08 ± 3.04)% in SGC-7901 cells compared to that of controls. Moreover, the proteins’ levels of Cleaved Caspase-3, Cleaved Caspase-9, and Cleaved PARP were significantly higher in C118P-treated HGC-27 and SGC-7901 cells than in controls, while Bax and Bcl-2 protein levels remained unchanged (Figure 3D–G). Collectively, these results demonstrate that C118P induces apoptosis of GC cells via a caspase-dependent intrinsic pathway.

### 3.4. C118P Inhibits GC Cells’ Growth via Targeting RAB1A

**Figure 4:**
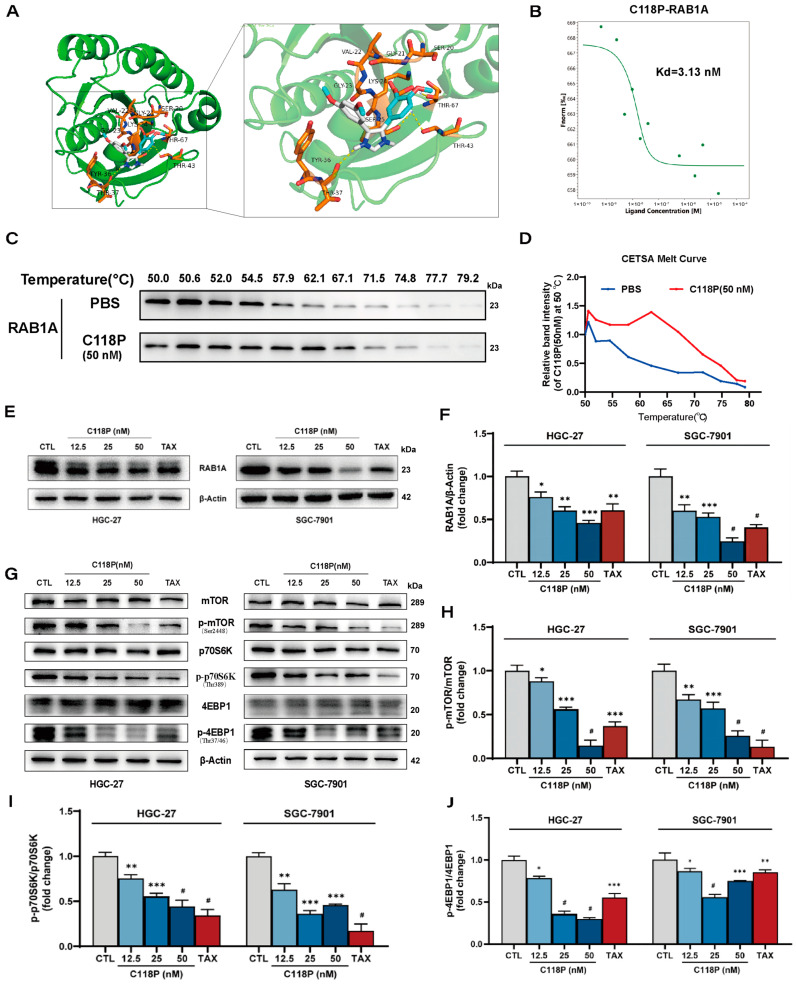
C118P inhibits the growth of GC cells via binding to RAB1A and inhibiting the RAB1A-mTOR axis. (**A**) Docked structure and interactions of C118P binding to RAB1A. (**B**) The representative dose–response curve of MST. (**C**,**D**) CETSA showing that C118P binds to RAB1A at the cellular level. A densitometry analysis of the bands from immunoblots was performed with ImageJ (Version 1.8.0.112) and normalized to that at 50 °C, respectively. (**E**,**F**) Representative immunoblotting images (**E**) and summarized data (**F**) showing a decreased protein level of RAB1A in GC cell lines HGC-27 and SGC-7901 treated with C118P (12.5, 25, 50 nM) for 24 h. (**G**) Representative immunoblotting images of protein expressions of mTOR, p-mTOR, p70S6K, p-p70S6K, 4EBP1, and p-4EBP1 in HGC-27 and SGC-7901 cells treated with C118P (12.5, 25, 50 nM) for 24 h. (**H**–**J**) Summarized data from the immunoblotting assay showing decreased protein expressions of p-mTOR (**H**), p-p70S6K (**I**), and p-4EBP1 (**J**) in HGC-27 and SGC-7901 cells treated with C118P (12.5, 25, 50 nM) for 24 h. Data are represented as the mean ± SD of three independent experiments. * *p* < 0.05, ** *p* < 0.01, *** *p* < 0.001, ^#^ *p* < 0.0001 vs. control group.

To explore the potential target of C118P, we performed molecular docking, MST assays, and a cellular thermal shift assay (CETSA). Reverse docking predicted that C118P could bind with RAB1A. The binding sites of C118P and RAB1A were SER-20, GLY-21, VAL-22, GLY-23, LYS-24, SER-25, TYR-36, THR-27, THR-43, and THR-67 (Figure 4A). By using MST, we further confirmed that C118P directly binds to RAB1A with a Kd value of 3.13 nM (Figure 4B). Consistently, the results from CETSA showed that C118P (50 nM) increased the thermal stabilization of RAB1A protein in the lysates of gastric cancer cells, suggesting that C118P binds to RAB1A in the cellular context (Figure 4C,D).

We next determined whether C118P exhibits regulatory effects on RAB1A in GC cells by measuring the expression and downstream pathway of RAB1A. As shown in Figure 4E,F, C118P (12.5, 25, 50 nM) markedly reduced the RAB1A protein level in a dose-dependent manner in both HGC-27 and SGC-7901 cells compared to the controls, while it exerted no effect on RAB1A mRNA levels (Figure S2). In addition, C118P treatment markedly inhibited the mTORC1 signaling pathway, which is downstream of RAB1A, as evidenced by a decreased phosphorylation expression of p-mTOR (Ser2448), p-p70S6K (Thr389), and p-4EBP1 (Thr37/46) in both HGC-27 and SGC-7901 cells compared to the controls (Figure 4G–J). Taken together, these results indicate that C118P represses growth of GC cells via inhibiting the activation of the RAB1A-mTOR axis.

### 3.5. Knockdown of RAB1A Attenuates Anti-GC Effect of C118P

**Figure 5:**
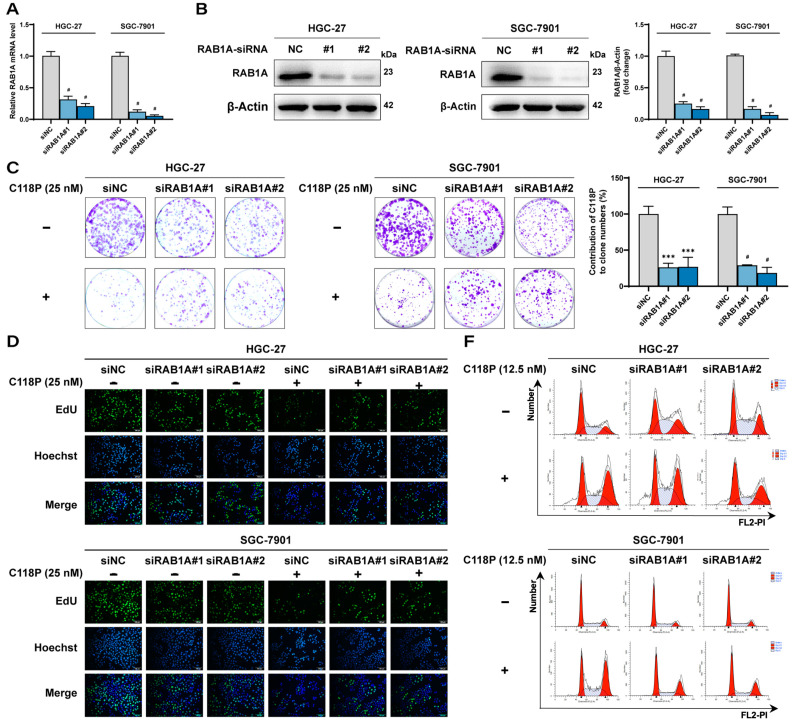

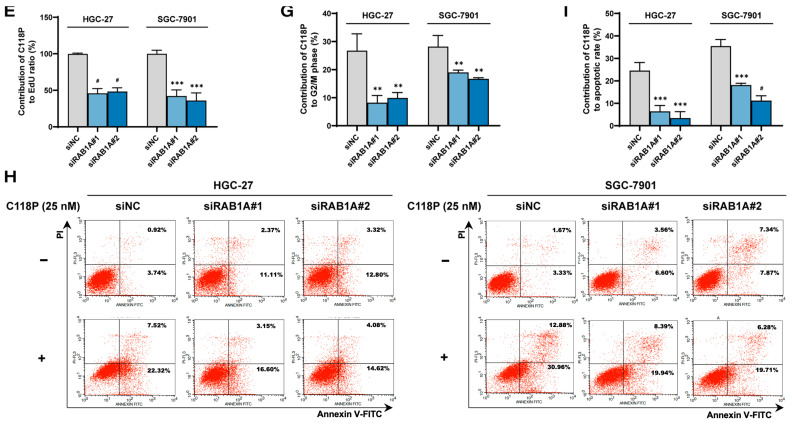
Effects of C118P on the growth of GC cell lines HGC-27 and SGC-7901 following RAB1A knockdown. (**A**) Summarized quantitative RT-PCR data showing a decreased RAB1A mRNA level in HGC-27 and SGC-7901 cells transfected with RAB1A-siRNA for 48 h compared to negative control siRNA. (**B**) Representative immunoblotting images (**left and middle panel**) and summarized data (**right panel**) showing a decreased protein expression of RAB1A in HGC-27 and SGC-7901 cells transfected with RAB1A-siRNA for 72 h compared to negative control siRNA (siNC). (**C**) Representative images (**left and middle panel**) of the colony formation assay showing cell density in HGC-27 and SGC-7901 cells treated with C118P (25 nM) after RAB1A knockdown and colony formation rates (**right panel**) were analyzed. (**D**,**E**) The cell proliferation of HGC-27 and SGC-7901 cells treated with C118P (25 nM) after RAB1A knockdown was tested by the EdU assay. Representative images (**D**) were photographed by an inverted fluorescence microscope and EdU ratios (**E**) were analyzed. Scale bar, 100 μm. (**F**,**G**) Representative images (**F**) and summarized data (**G**) of the flow cytometry assay showing the cell cycle distribution of HGC-27 and SGC-7901 cells treated with C118P (12.5 nM) after RAB1A knockdown. (**H**,**I**) Representative images (**H**) and summarized data (**I**) of the flow cytometry assay (Annexin V/PI) showing the apoptotic cells in HGC-27 and SGC-7901 cells treated with C118P (25 nM) after RAB1A knockdown. Data are represented as the mean ± SD of three independent experiments. ** *p* < 0.01, *** *p* < 0.001, ^#^ *p* < 0.0001 vs. negative control group.

To further determine the contribution of RAB1A to the anti-GC effect of C118P, we detected the effect of C118P on malignant phenotypes in GC cells after RAB1A knockdown using short interfering RNA fragments of RAB1A (siRAB1A). Both siRAB1A#1 and siRAB1A#2 effectively reduced RAB1A mRNA and protein expression to near-undetectable levels in HGC-27 and SGC-7901 cell lines compared to the negative control (Figure 5A,B). RAB1A knockdown significantly inhibited cell proliferation, and induced apoptosis and cell cycle arrest in HGC-27 and SGC-7901 cells (Figure 5C,D,H and Figure S3A). Notably, RAB1A knockdown significantly mitigated C118P’s anti-proliferative effect, with a ~3-fold reduction in relative inhibition in colony formation assays (Figure 5C and Figure S3A). Similar results were observed in EdU staining assays (Figure 5D,E and Figure S3B). Furthermore, the G2/M arrest induced by C118P was also diminished in RAB1A-depleted cells (Figure 5F,G and Figure S3C). Additionally, the knockdown of RAB1A significantly decreased the pro-apoptotic effect of C118P in HGC-27 and SGC-7901 cells (Figure 5H,I and Figure S3D). Collectively, these results demonstrated that the anti-tumor activities of C118P against GC cells were attenuated following RAB1A knockdown, reflecting that C118P exerts its anti-tumor effects in GC cells via targeting RAB1A.

### 3.6. Overexpression of RAB1A Sensitizes GC Cells to C118P Treatment

**Figure 6:**
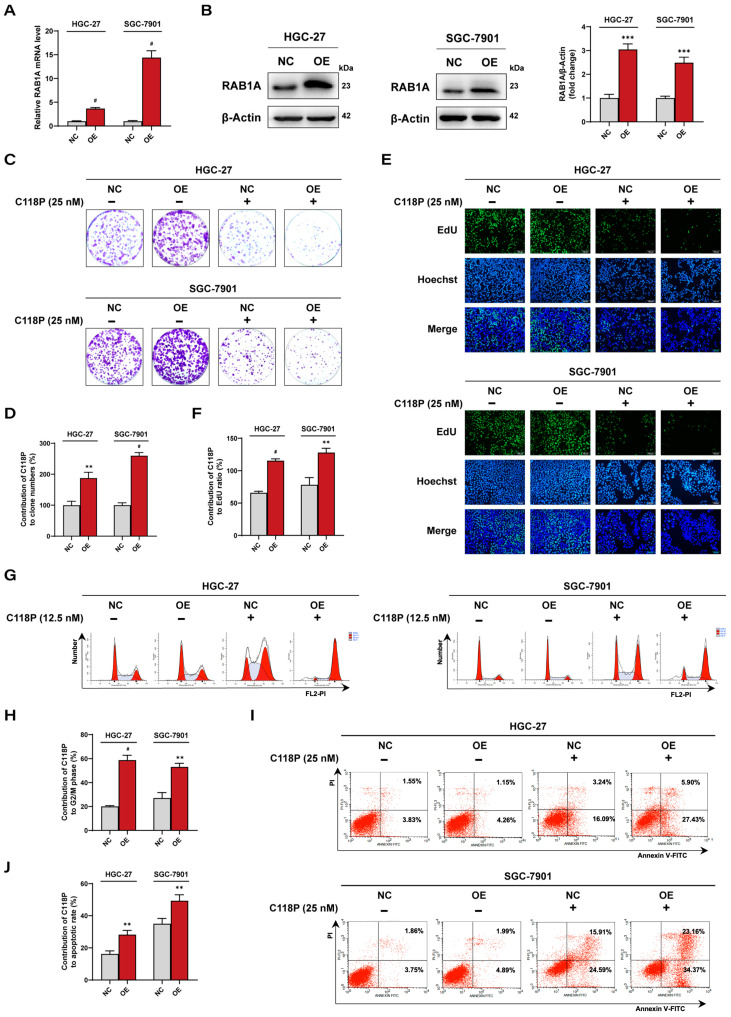
Effects of C118P on the growth of GC cell lines HGC-27 and SGC-7901 following RAB1A overexpression. (**A**) Summarized quantitative RT-PCR data showing an increased RAB1A mRNA level in HGC-27 and SGC-7901 cells transfected with a DNA–lipid complex expressing RAB1A (OE) vs. negative control (NC). (**B**) Representative immunoblotting images (**left and middle panel**) and summarized data (**right panel**) showing an increased protein level of RAB1A in HGC-27 and SGC-7901 cells transfected with a DNA–lipid complex expressing RAB1A (OE) compared to negative control (NC). (**C**,**D**) Representative images (**C**) of the colony formation assay showing cell density in GC cell lines HGC-27 (**upper panel**) and SGC-7901 (**lower panel**) treated with C118P (25 nM) after RAB1A overexpression and colony formation rates (**D**) were analyzed. (**E**,**F**) The cell proliferation of HGC-27 and SGC-7901 cells treated with C118P (25 nM) after RAB1A overexpression was tested by the EdU assay. Representative images (**E**) were photographed by an inverted fluorescence microscope and EdU ratios (**F**) were analyzed. Scale bar, 100 μm. (**G**,**H**) Representative images (**G**) and summarized data (**H**) of the flow cytometry assay showing the cell cycle distribution of HGC-27 and SGC-7901 cells treated with C118P (12.5 nM) after RAB1A overexpression. (**I**,**J**) Representative images (**I**) and summarized data (**J**) of the flow cytometry assay (Annexin V/PI) showing the apoptotic cells in HGC-27 and SGC-7901 cells treated with C118P (25 nM) after RAB1A overexpression. Data are represented as the mean ± SD of three independent experiments. ** *p* < 0.01, *** *p* < 0.001, ^#^ *p* < 0.0001 vs. negative control group.

We next evaluated the effect of C118P on malignant phenotypes in GC cells with RAB1A overexpression. Following transfection with RAB1A DNA–lipid complexes, GC cells successfully overexpressed RAB1A at both mRNA and protein levels (Figure 6A,B). Consistent with RAB1A’s role in promoting tumor malignancy, RAB1A overexpression significantly stimulated GC cell growth (Figure 6C,E and Figure S4A,B). Strikingly, the anti-proliferative effect of C118P was markedly enhanced in GC cells overexpressing RAB1A compared to the negative control (NC) group (Figure 6D,F). Moreover, the influence of C118P on cell cycle arrest was augmented in RAB1A-overexpressing GC cells, evidenced by the higher relative frequency of GC cells in the G2/M phase in the C118P-treated RBA1A OE GC cells than that of NC GC cells (Figure 6G,H and Figure S4C). Additionally, C118P treatment elicited a higher rate of apoptosis in HGC-27 and SGC-7901 cells with overexpressing RAB1A compared to the NC group, suggesting that the pro-apoptotic action of C118P was reinforced in GC cells with higher RAB1A levels (Figure 6I,J and Figure S4D). Taken together, these findings demonstrated that RAB1A is critical for C118P’s anti-tumor activity in GC cells, highlighting its role as a key therapeutic target.

### 3.7. C118P Promotes Autophagic–Lysosomal Degradation of RAB1A in GC Cells

We next explored how C118P reduces RAB1A expression in GC cells. Given that C118P induces a decrease in the RAB1A protein level rather than the mRNA level, we concentrated on the function of C118P in regulating RAB1A protein expression via protein synthesis and protein degradation. As shown in Supplementary Figure S5A–D, C118P treatment further reduced RAB1A protein expression in the presence of the translation inhibitor, CHX, compared to cells treated with CHX alone, suggesting that C118P had little impact on RAB1A protein synthesis. In eukaryotic cells, protein degradation is mainly mediated by the ubiquitin-proteasome pathway and autophagy–lysosomal pathways. Our studies demonstrated that C118P-mediated RAB1A protein downregulation may not rely on the ubiquitin-proteasome pathway, as its inhibition by proteasome inhibitor MG132 could not reverse the decreased RAB1A protein seen in C118P-treated GC cells (Figure S5E,F).

**Figure 7:**
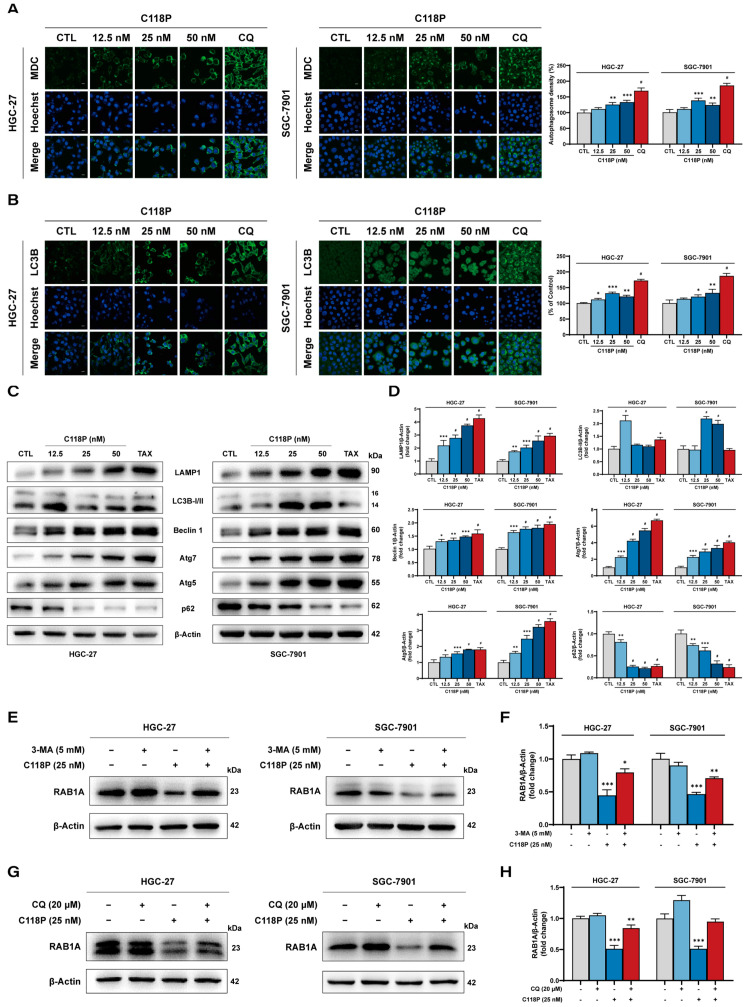
Effects of C118P on the autophagic–lysosomal degradation of RAB1A in GC cell lines HGC-27 and SGC-7901. (**A**) The eosinophilic vacuoles in HGC-27 (left panel) and SGC-7901 (**middle panel**) cells treated with C118P (12.5, 25, 50 nM) for 24 h were labeled by MDC, and autophagosome density (**right panel**) was analyzed. Scale bar, 40 μm. (**B**) The formation of autophagosomes in HGC-27 (**left panel**) and SGC-7901 (**middle panel**) cells treated with C118P (12.5, 25, 50 nM) for 24 h was detected by immunofluorescence staining of LC3B, and endogenous LC3B puncta per cell (**right panel**) were analyzed. CQ (20 μM) was used as the positive control. Scale bar, 40 μm. (**C**,**D**) Representative immunoblotting images (**C**) and summarized data (**D**) showing the protein expression of LAMP1, LC3B-I/II, Beclin 1, Atg7, Atg5, and p62 in HGC-27 and SGC-7901 cells treated with C118P (12.5, 25, 50 nM) for 24 h. (**E**,**F**) Representative immunoblotting images (**E**) and summarized data (**F**) showing the protein level of RAB1A in HGC-27 and SGC-7901 cells treated with 3-MA (5 mM) and C118P (25 nM) for 24 h. (**G**,**H**) Representative immunoblotting images (**G**) and summarized data (**H**) showing the protein level of RAB1A in HGC-27 and SGC-7901 cells treated with CQ (20 μM) and C118P (25 nM) for 24 h. Data are represented as the mean ± SD of three independent experiments. * *p* < 0.05, ** *p* < 0.01, *** *p* < 0.001, ^#^ *p* < 0.0001 vs. control group.

To determine whether C118P promoted the degradation of RAB1A protein via an autophagy–lysosomal pathway, we first assessed the role of C118P in autophagy by observing autophagosomes using fluorescent probes MDC and LC3B. Interestingly, the results from MDC staining showed that C118P treatment induced an abundance of clear punctate structures in the cytoplasm or perinucleus of HGC-27 and SGC-7901 cells (Figure 7A), suggesting that C118P induced autophagosome formation in GC cells. Moreover, Figure 7B also shows an increase in the number of endogenous LC3B puncta in the cytoplasm of GC cells treated with C118P compared to that of controls, indicative of autophagosome accumulation. To probe this further, we performed immunoblotting assays to detect the protein expressions of numerous autophagy/lysosomal-related markers, and found that C118P treatment increased the expression of the autophagy markers LC3B-II, Beclin 1, Atg7, and Atg5 and lysosomal marker LAMP1. Conversely, p62 protein levels were dramatically decreased in a dose-dependent manner in HGC-27 and SGC-7901 cells treated with C118P (Figure 7C,D). Thus, these results demonstrate that C118P promotes the occurrence of autophagy and the activation of the lysosomal pathway in GC cells.

Furthermore, autophagy inhibitor 3-MA and lysosomal inhibitor CQ were employed to confirm whether C118P promoted RAB1A protein degradation via the autophagy–lysosomal pathway. Figure 7E–H show that either autophagy inhibition by 3-MA or lysosomal inhibition by CQ reversed C118P-mediated RAB1A protein downregulation in GC cells compared with C118P treatment alone. Taken together, our results demonstrate that C118P causes RAB1A protein degradation via the autophagy–lysosomal pathway in gastric cancer.

### 3.8. C118P Exhibits an Anti-GC Effect In Vivo

**Figure 8:**
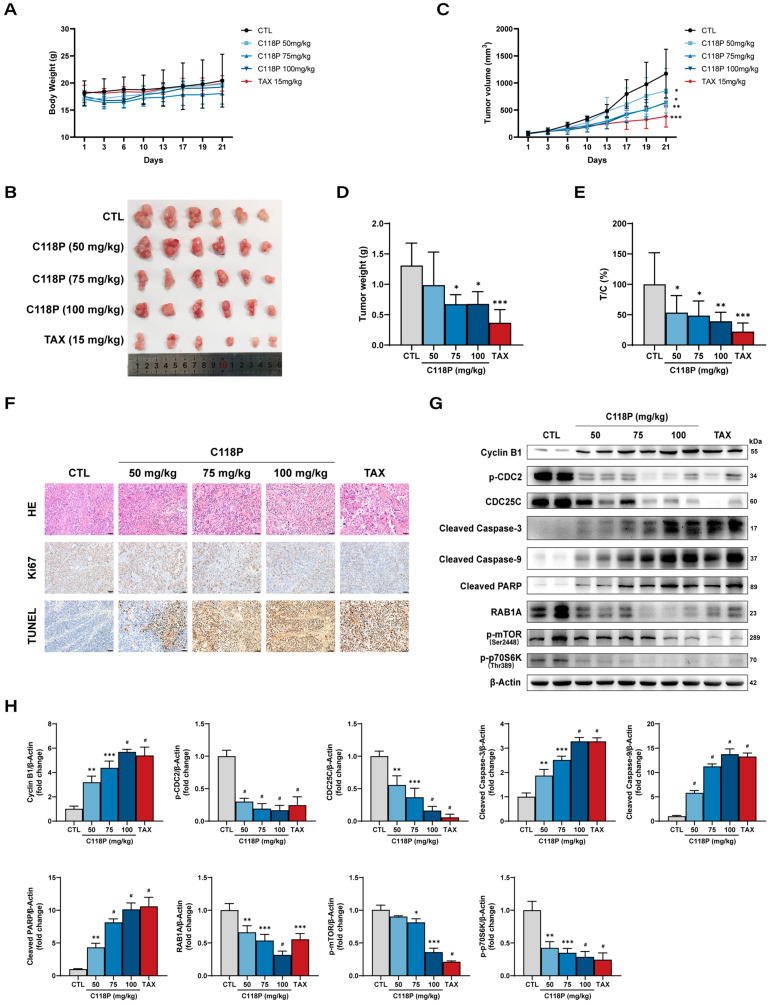
The effect of C118P on SGC-7901 xenograft growth in vivo. (**A**) Body weight of tumor-bearing mice. (**B**) An image of resected tumors in tumor-bearing mice treated with C118P (50, 75, 100 mg/kg) and Taxol (TAX, 15 mg/kg). (**C**–**E**) Summarized data showing tumor volume (**C**), tumor weight (**D**), and T/C (**E**) of tumor-bearing nude mice treated with C118P (50, 75, 100 mg/kg) and Taxol (TAX, 15 mg/kg). (**F**) Representative images of hematoxylin and eosin staining and immunohistochemistry of Ki67- and TUNEL-positive cells in the tumor tissues of tumor-bearing nude mice treated with C118P (50, 75, 100 mg/kg) and Taxol (TAX, 15 mg/kg). Scale bar, 50 μm. (**G**,**H**) Representative immunoblotting images (**G**) and summarized data (**H**) showing the protein expression of Cyclin B1, p-CDC2, CDC25C, Cleaved Caspase-3, Cleaved Caspase-9, Cleaved PARP, RAB1A, p-mTOR, and p-p70S6K in the tumor tissues of tumor-bearing nude mice treated with C118P (50, 75, 100 mg/kg) and Taxol (TAX, 15 mg/kg). Taxol was used as the positive control. n = 6 per group. Data are represented as the mean ± SD. * *p* < 0.05, ** *p* < 0.01, *** *p* < 0.001, ^#^ *p* < 0.0001 vs. control group.

The anti-tumor effects of C118P against gastric cancer were validated in vivo using an SGC-7901 xenograft mouse model. Firstly, we did not observe significant toxicity of C118P, reflected in unchanged body weight at all three concentrations compared to the control (Figure 8A). Remarkably, C118P (50, 75, or 100 mg/kg) or Taxol (15 mg/kg) administration attenuated tumor growth in tumor-bearing nude mice compared to the controls (Figure 8B). The overall size and weight of tumors in the C118P-treated groups were significantly lower than those in the negative control group (Figure 8C,D). Figure 8E of the analysis of tumor volume revealed relative tumor proliferation rates (T/C%) of 52.10%, 42.84%, and 42.37% in mice treated with C118P at 50, 75, and 100 mg/kg, respectively, and 25.10% inhibition in mice treated with Taxol (15 mg/kg) (Figure 8E). The effects of C118P on GC cell proliferation and apoptosis in vivo were confirmed through HE staining and immunohistochemical staining detecting Ki67- and TUNEL-positive cells. As shown in Figure 8F, Ki67 expression was markedly reduced in C118P-treated tumors compared to that of negative controls, indicating that C118P suppresses the tumor proliferation in vivo. Meanwhile, the TUNEL staining results showed that C118P dramatically increased the proportion of TUNEL-positive cells in tumor tissues, indicating the pro-apoptosis effect of C118P in vivo. Indeed, the results of immunoblotting further confirmed that C118P inhibited GC tumor growth in vivo via G2/M cell cycle arrest and apoptosis, which is consistent with the in vitro results as shown in Figure 2C–F and Figure 3D–G.

In addition, the effect of C118P on the RAB1A-mTOR pathway in vivo was verified by the immunoblotting analysis detecting key components of the RAB1A-mTOR pathway. In line with the inhibition of the mTORC1 pathway by C118P administration in vitro as shown in Figure 4, a clear reduction in the protein expression of RAB1A, p-mTOR, and p-p70S6K was observed in the tumor tissues of C118P-treated mice compared to the negative controls, suggesting the inhibitory effect of C118P on the RAB1A-mTOR axis in vivo (Figure 8G,H). Taken together, our data demonstrate that C118P suppresses the growth of tumors via inhibiting proliferation and inducing apoptosis, as well as targeting the RAB1A-mTOR axis in gastric cancer.

## 4. Discussion

In the current study, we observed that C118P could inhibit GC growth via inducing G2/M-phase cell cycle arrest and apoptosis both in vitro and in vivo. In this context, C118P exhibited its anti-neoplastic actions through directly binding to RAB1A and inhibiting RAB1A-mediated mTORC1 signaling. Furthermore, our data revealed that C118P induced autophagosome formation and promoted RAB1A protein degradation via the autophagy–lysosomal pathway.

Numerous investigations have provided evidence that abnormal RAB1A expression is a common occurrence in human cancers, such as colorectal cancer [21], GC [23], bladder cancer [24], HCC [34], breast cancer [35], and nasopharyngeal cancer [36]. RAB1A is involved in tumor progression, including the cell cycle, proliferation, and apoptosis, and has been shown to promote tumor growth during carcinogenesis and progression in gastric cancer [37]. The overexpression of RAB1A promotes oncogenesis and makes cancer cells vulnerable to mTORC1-targeted treatment [25]. In our study, C118P was demonstrated to bind to RAB1A with high affinity and thereby regulate RAB1A expression at the post-transcriptional translational level. Additionally, the mTORC1 signaling pathway was also significantly inhibited by C118P treatment both in in vivo and in vitro studies. By using gain-of-function and loss-of-function approaches, we found that the contribution of C118P to inhibiting GC cell growth was weakened in the setting of RAB1A knockdown. Conversely, RAB1A overexpression sensitized GC cells to C118P treatment.

Recent studies have shown that RAB1A is primarily regulated by long non-coding RNAs and microRNAs at the post-transcriptional level [27,38,39]. Our data showed that C118P further reduced RAB1A protein expression in the presence of the protein synthesis inhibitor CHX, implying that C118P may reduce RAB1A protein levels via the protein degradation pathway. The protein degradation system, essential for maintaining cellular homeostasis, is often dysregulated in diseases such as Parkinson’s disease, Alzheimer’s disease, and cancer [40]. The ubiquitin-proteasome pathway (UPP) and autophagy–lysosomal pathway (ALP) are two major intracellular protein degradation pathways [41]. Although RAB1A was shown to be regulated by Hsc70 via the ubiquitin-proteasome pathway (UPP) [42], our research revealed that it was not the way that C118P mediated RAB1A protein degradation in this context. Autophagy is essential for all eukaryotic cells to maintain cellular homeostasis, which explains why both normal and malignant cells benefit from autophagic response. Autophagy maturation is defined as the fusion of autophagosomes and lysosomes to form autophagolysosomes, which results in the degradation of wrapped contents in the presence of hydrolases in the lysosomes [43,44]. Autophagy-related proteins play crucial roles in the dynamic process of autophagy. Beclin 1 [45], LC3B [46], Atg [47], and p62 [48] are all momentous regulators in the autophagic process, while LAMP1 serves as a marker for lysosomal activity [49,50]. In this study, we found that C118P promoted autophagosome formation in GC cells, and upregulated autophagy-related proteins (LC3B-II, Beclin 1, Atg7, and Atg5) and LAMP1. Furthermore, using pharmacological inhibitors of autophagy (3-MA) and lysosomal activity (CQ), we confirmed that C118P facilitates RAB1A protein degradation through the autophagy–lysosomal pathway.

C118P, a small molecule compound with anti-angiogenic and vascular-disrupting activities, is a promising chemotherapeutic agent for solid tumors [30]. However, cancer therapy faces challenges due to patient and tumor heterogeneity, which limits the effectiveness of chemotherapy. For instance, paclitaxel, a well-established chemotherapeutic agent for cancer, is not effective in all patients and can cause significant side effects [51,52]. This underscores the need to improve the application of C118P in cancer treatment. Our current study showed that C118P has a considerable inhibitory effect on gastric cancer via targeting RAB1A, suggesting its potential for cancers with high RAB1A expression. Future investigation using gastric cancer organoids will further validate these findings. Additionally, combining gene-targeted therapies with immunotherapies offers a promising strategy to overcome the complexity of tumor biology. CAR T-cell therapy, which modifies T-cells to recognize the antigens expressed on malignant cells, is being evaluated in solid tumors [53,54]. The vascular-disrupting properties of C118P may complement CAR T-cells by improving tumor penetration and efficacy [30,55].

## Data Availability

Data is contained within the article or Supplementary Material.

## Supplementary Materials

The following supporting information can be downloaded at: www.mdpi.com/10.3390/pharmaceutics16121620/s1, Figure S1: The Structural formula of C118P (http://www.sanhome.com/en/inside/45/103.html); Figure S2: Effect of C118P on the mRNA expression of GC cell lines HGC-27 and SGC-7901; Figure S3: Effects of C118P on the growth of GC cell lines HGC-27 and SGC-7901 following RAB1A knockdown; Figure S4: Effects of C118P on the growth of GC cell lines HGC-27 and SGC-7901 following RAB1A overexpression; Figure S5: Effect of CHX or MG132 on C118P-mediated down-regulation of RAB1A protein expression in GC cell lines HGC-27 and SGC-7901.
