# Supplementary material for: C118P Suppresses Gastric Cancer Growth via Promoting Autophagy–Lysosomal Degradation of RAB1A
Source: Pharmaceutics. 2024 Dec 21;16(12):1620. doi: 10.3390/pharmaceutics16121620 (PMC11678531; doi:10.3390/pharmaceutics16121620)
Supplement: Supplementary file 1 [file pharmaceutics-16-01620-s001.zip › pharmaceutics-3361558-supplementary.pdf]

Supplemental Information

Supplemental_Figure S1

A

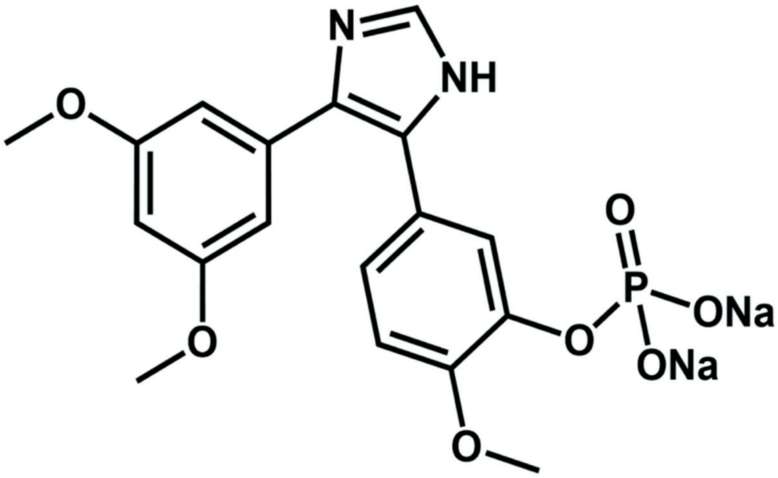

Figure S1 Chemical Structure of C118P (http://www.sanhome.com/en/inside/45/103.html)

Supplemental_Figure S2

A

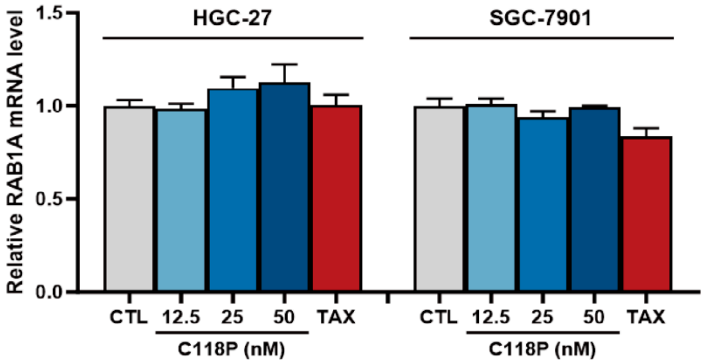

Figure S2 Effect of C118P on the mRNA expression of GC cell lines HGC-27 and SGC-7901. (A) Summarized quantitative RT-PCR data showing no significant changes of RAB1A mRNA level in HGC-27 and SGC-7901 cells treated with C118P (12.5, 25, 50 nM) for 24 h.

Supplemental_Figure S3

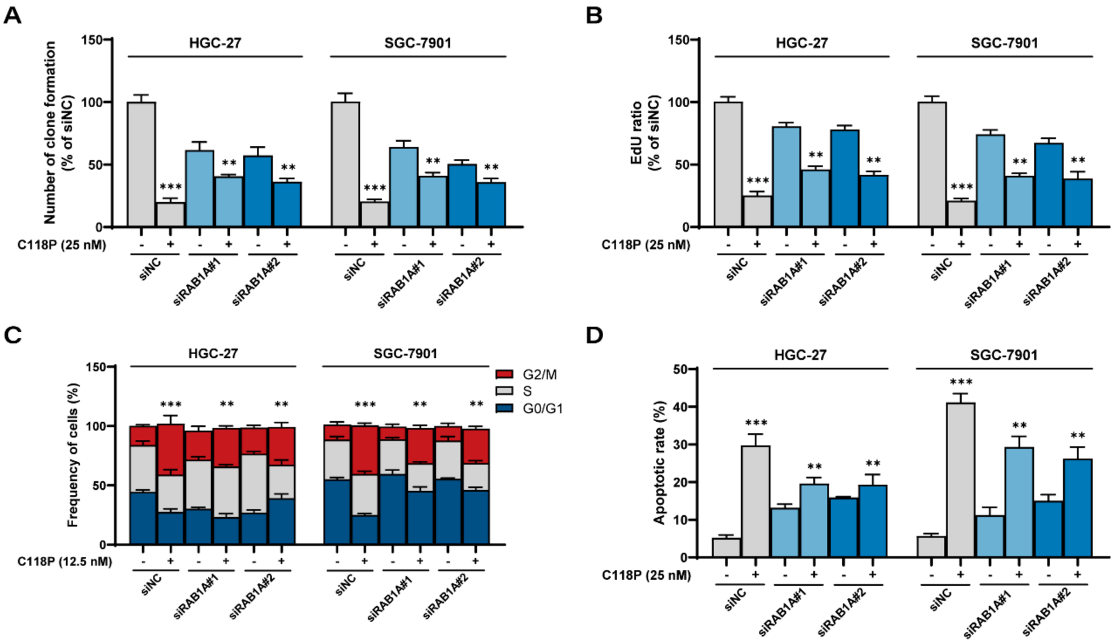

Figure S3 Effects of C118P on the growth of GC cell lines HGC-27 and SGC-7901 following RAB1A knockdown. (A) Colony formation rates of HGC-27 and SGC-7901 cells treated with C118P (25 nM) after RAB1A knockdown were analyzed. (B) EdU ratios of HGC-27 and SGC-7901 cells treated with C118P (25 nM) after RAB1A knockdown were analyzed. (C) Cell cycle distribution of HGC-27 and SGC-7901 cells treated with C118P (12.5 nM) after RAB1A knockdown was analyzed. (D) Apoptosis rates of HGC-27 and SGC-7901cells treated with C118P (25 nM) after RAB1A knockdown were analyzed. Data are represented as mean ± SD of three independent experiments. **p*<0.05, ***p*<0.01, ****p*<0.001, #*p*<0.0001 *vs*. negative control group.

Supplemental_Figure S4

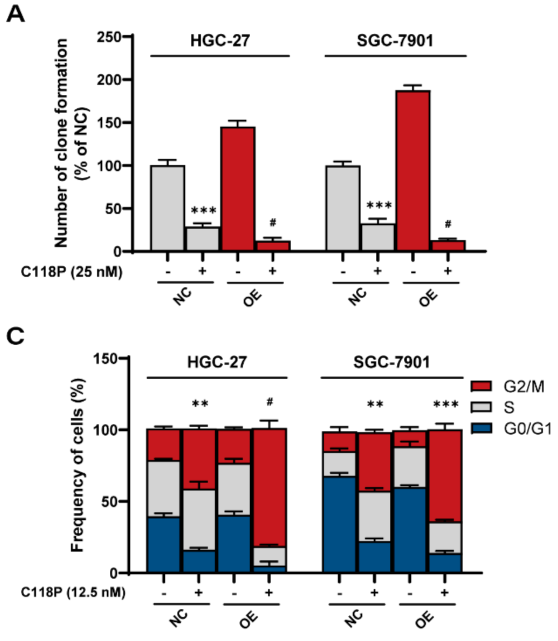

Figure S4 Effects of C118P on the growth of GC cell lines HGC-27 and SGC-7901 following RAB1A overexpression. (A) Colony formation rates of HGC-27 and SGC-7901 cells treated with C118P (25 nM) after RAB1A overexpression were analyzed. (B) EdU ratios of HGC-27 and SGC-7901 cells treated with C118P (25 nM) after RAB1A overexpression were analyzed. (C) Cell cycle distribution of HGC-27 and SGC-7901 cells treated with C118P (12.5 nM) after RAB1A overexpression was analyzed. (D) Apoptosis rates of HGC-27 and SGC-7901cells treated with C118P (25 nM) after RAB1A overexpression were analyzed. Data are represented as mean ± SD of three independent experiments. $*p<0.05$, $**p<0.01$, $***p<0.001$, $\#p<0.0001$ *vs*. negative control group.

Supplemental_Figure S5

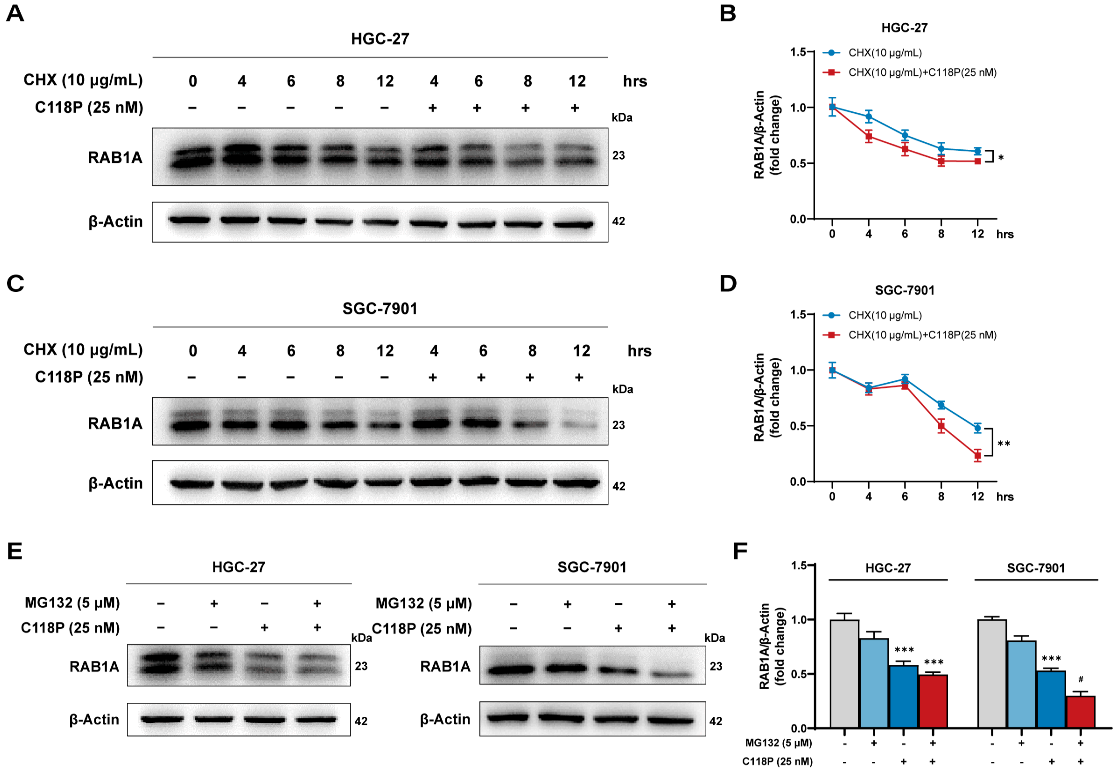

Figure S5 Effect of CHX or MG132 on C118P-mediated down-regulation of RAB1A protein expression in GC cell lines HGC-27 and SGC-7901. (A-B) Representative immunoblotting images (A) and summarized data (B) showing the protein level of RAB1A in HGC-27 cells treated with CHX (10 μg/mL) and C118P (25 nM) for 24 h. (C-D) Representative immunoblotting images (C) and summarized data (D) showing the protein level of RAB1A in SGC-7901 cells treated with CHX (10 μg/mL) and C118P (25 nM) for 24 h. (E-F) Representative immunoblotting images (E) and summarized data (F) showing the protein level of RAB1A in HGC-27 and SGC-7901 cells treated with MG132 (5 μM) and C118P (25 nM) for 24 h. Data are represented as mean ± SD of three independent experiments. **p*<0.05, ***p*<0.01, ****p*<0.001, #*p*<0.0001 *vs*. control group.
